# Tableware Tidying-Up Robot System for Self-Service Restaurant–Detection and Manipulation of Leftover Food and Tableware-

**DOI:** 10.3390/s22187006

**Published:** 2022-09-15

**Authors:** Deheng Zhu, Hiroaki Seki, Tokuo Tsuji, Tatsuhiro Hiramitsu

**Affiliations:** 1 Division of Mechanical Science and Engineering, Kanazawa University, Kakuma, Kanazawa 920-1192, Ishikawa, Japan; 2 Institute of Science and Engineering, Kanazawa University, Kakuma, Kanazawa 920-1192, Ishikawa, Japan

**Keywords:** parallel arm, leftover food treatment, tableware, tidying-up robot, RGB-D camera, self-service restaurant, air cylinder

## Abstract

In this study, an automated tableware tidying-up robot system was developed to tidy up tableware in a self-service restaurant with a large amount of tableware. This study focused on sorting and collecting tableware placed on trays detected by an RGB-D camera. Leftover food was also treated with this robot system. The RGB-D camera efficiently detected the position and height of the tableware and whether there was leftover food or not by image processing. A parallel arm and robot hand mechanism was designed to realize the advantages of a low cost and high processing speed. Two types of rotation mechanisms were designed to realize the function of throwing away leftover food. The effectiveness of the camera detection system was verified through the experiments of tableware and leftover food detection. The effectiveness of the prototype robot and the rotation assist mechanism was verified through the experiments of grasping tableware, throwing away leftover food by two types of rotating mechanisms, collecting multiple tableware, and the sorting of overlapping tableware with multiple robots.

## 1. Introduction

In recent years, the labor shortage has become a major challenge with the advent of an aging society. Robots are expected to be introduced to automate human tasks. The chain in the meal industry includes food production, food provision, table cleaning, tableware sorting, and cleaning. Some studies designed a two-handed robot for cooking, expanding the motion sparseness, optimizing the mission, and shortening the cooking time [1,2]. Some studies focusing on food provision used multiple robots designed with a ringed robot by planning the robot’s running trajectory [3,4]. Other studies reported on the collection of tableware from a table by using a single motor to operate multiple fingers simultaneously and attaching a camera to grasp the overall condition of the tableware [5,6]. Some research on tableware sorting were also primarily conducted for homes with some tableware, for which the length of time does not matter. A six-axis serial robot with a hand with many sensors was developed to trace the tableware to grasp the state of the tableware and then put it into the washing machine [7]. In this study, it was reported to take 1 min 42 s to clean up one piece of tableware, which is time-consuming. High-speed processing is necessary to tidy up tableware, especially in a self-service restaurant environment where the quantity of tableware is large and the work is monotonous. Concerning these issues, this study focused on following the characteristics of tidying up tableware in a self-service restaurant, “After a person puts the tableware on the tray the amount of tableware is large, the type of tableware is limited, and the time to tidy up the tableware needs to be shortened”. The contribution of this study is that we proposed a method to detect overlapping tableware and leftover food using a combination of gray images and depth images that improved the reliability of detection. The proposed method can operate with a simple robot arm at a high speed and low cost.

The rest of the paper is organized as follows. Section 2 introduces the configuration of the high-speed, low-cost tableware tidying up system and explains the working of each part. Section 3 discusses image processing for detecting tableware and leftover food. Section 4 presents the design of each part of the robot and the tableware sorting robot system. The image processing experiments and the tableware sorting experiments by the robot system are discussed in Section 5. Section 6 presents a summary of the study, including future research directions.

## 2. Robot System Design

### 2.1. System Configuration

The processing goals of the system, mechanism, and dimensions of each part of the tableware are summarized in Table 1.

**Figure 1:**
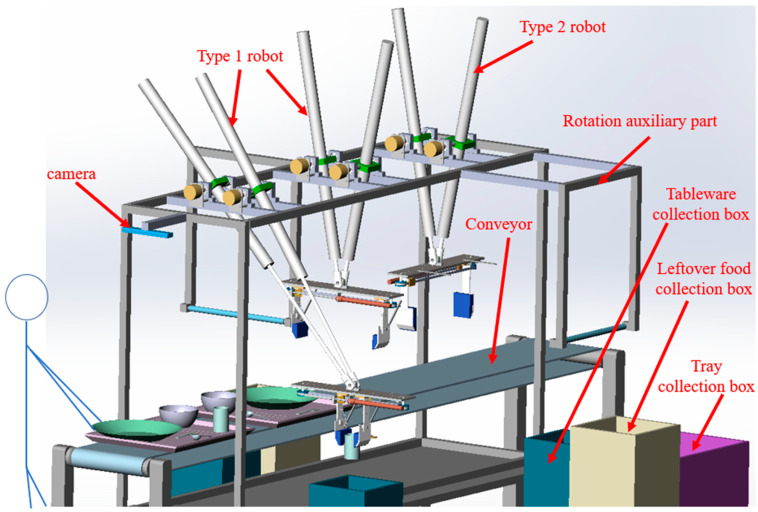
Overall view of the robot system.

We designed a high-speed tableware cleaning robot system to meet the goal as shown in Figure 1. This system mainly included a camera that detects the type and position of tableware and the presence or absence of leftover food, two types of robots that tidy up the tableware, an auxiliary mechanism that tilts the tableware to throw away the leftover food, a belt conveyor to flow the tray in which the tableware is placed, and some collection boxes for each tableware and tray.

A six-axis serial robot can realize the operation of tidying up the tableware, but it is not advantageous from the perspective of the processing speed and cost. It is better to use two air cylinders and a three-axis parallel robot because of the water and dirt when sorting up the tableware, and the accuracy of the cylinder can meet the target accuracy of the system. Many simple parallel robot arms can be lined up to achieve high speeds. The complete process includes grabbing the tableware, moving it, and putting the tableware in the collection box by expanding and contracting the air cylinder controlled by a five-port three-position closed center solenoid valve. The robot can grab the tableware with a hand that can control the opening and closing of two fingers simultaneously with one air cylinder.

**Figure 2:**
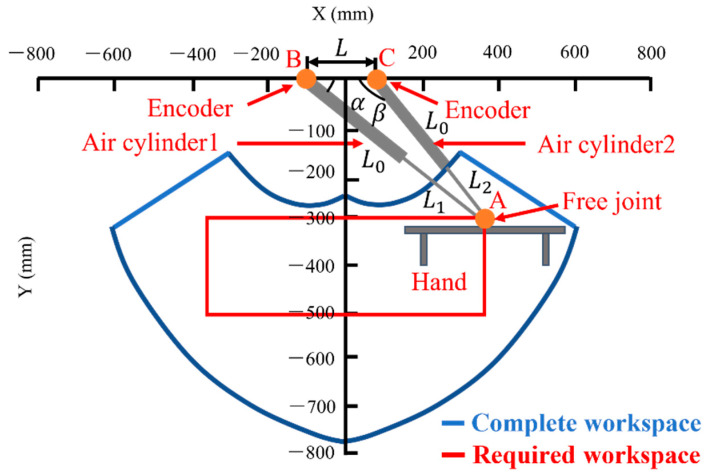
Structure and movable range of the parallel robot arm.

As shown in Figure 2, the three-axis parallel robot consists of a 2-DOF arm and a 1-DOF hand. Points B and C fix the air cylinder to the frame with a rotating shaft. The origin point is the midpoint between two rotating shafts. Because it is difficult to measure the stroke of an air cylinder directly by a linear sensor, rotary encoders are chosen and attached to axes B and C to measure α and β. The arm position can be controlled by calculated cylinder stroke using kinematics with α and β as shown in Equation (1).

(1) $$\begin{array}{l} X=L\left(\tan\beta -\tan\alpha \right)/2\left(\tan\beta +\tan\alpha \right)\\ Y=L\tan\alpha \tan\beta /\left(\tan\alpha +\tan\beta \right) \end{array}$$

Point A at the tip of the air cylinder is connected by a rotating shaft so that it can rotate. The approximate workspace of the robot arm is set as 800 mm × 200 mm. When the dimensions of the robot arm were determined to satisfy this condition, the stroke of the air cylinder was 500 mm, the initial length was $L_{0}$ = 270 mm, and the distance between the rotating shafts was L = 180 mm. The blue frame indicates the movable range of the robot. It describes the positions where the strokes of the two air cylinders expand and contract by 20 mm. The red frame is the target range that satisfies the experimental requirements.

### 2.2. Rotation Structure and Design

**Figure 3:**
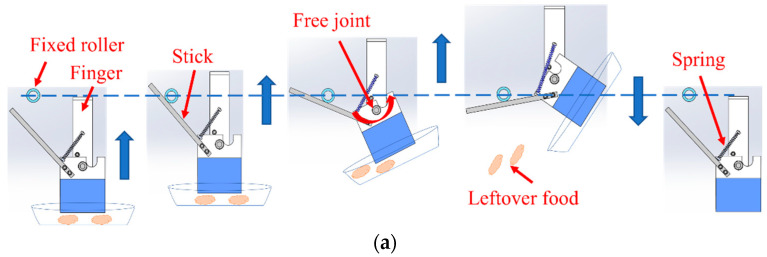

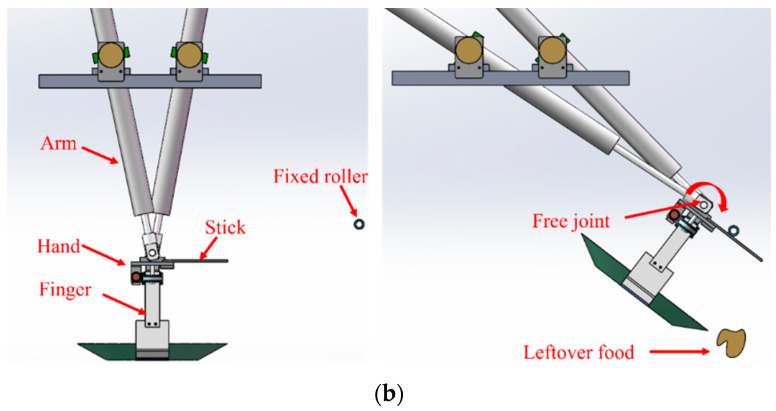
Flow of throwing away leftover food by two types of robots: (**a**) flow of throwing away leftover food with type 1 robot; (**b**) flow of throwing away leftover food with type 2 robot.

Two-hand design plans for tilting the tableware were built to throw away the leftover food. The fingers opening and closing are similar to the two types of hands. Two fingers are attached to the linear guide and connected to the timing belt so that they can open and close simultaneously. The timing belt is driven by an air cylinder to control the opening and closing of two fingers at the same time. Sponges are attached to the fingers so that the tableware can easily grasped with a larger frictional force [8]. The main difference is the design of the parts to throw away leftover food, illustrated in Figure 3. In type 1, a rotation axis in the direction of the air cylinder is provided in the middle of the finger so that half of the fingertip can rotate. A stick is attached to the rotating part, hooked on a roller fixed to the frame, and the arm raised the hand to rotate it. In addition, a spring is attached to the rotating fingertip to return it to its original position. In type 2, the T-shaped bearing is rotated 90 degrees and connected to the arm. Two rods are attached to the board of the hand, and by hitting the fixed roller, the tableware grabbed by the hands is tilted, and the leftover food is thrown away. The designed mechanism that rotates the tableware without using a motor could reduce the weight and cost of the hand [9].

## 3. Image Processing

### 3.1. Overall Flow of Image Processing

To tidy up the tableware, it was necessary to detect the type, distance, position, and leftover food on the tableware. Therefore, one RGB-D camera was used to capture gray and depth images [10,11,12]. The target tableware are plates, bowls, and cups. The RGB-D camera used the diameter and height of the tableware to distinguish the tableware and to detect leftover food and overlapping tableware.

**Figure 4:**
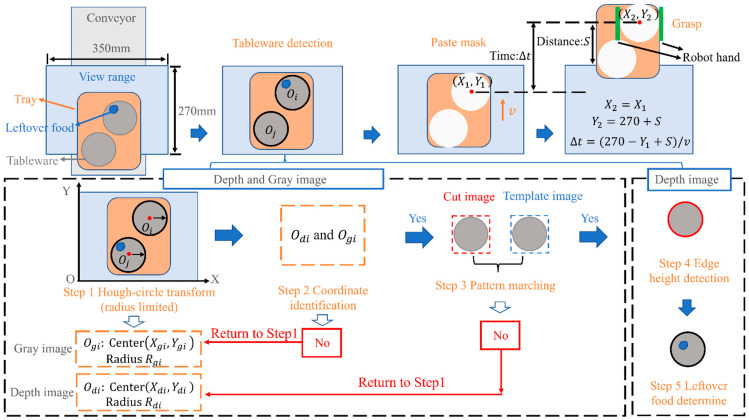
Overall flow of image processing.

As shown in Figure 4, the image processing begins when the tableware placed on the tray flows into the range of the camera by conveyor. Both the gray and depth images determine the position of the tableware. First, the position and radius of the tableware are detected with the Hough circle transform [13,14,15,16,17,18,19], which enables to check whether the coordinates detected in the gray image and the depth image are the same, aiming to prevent erroneous detection. If they are detected not the same, the process returns to the Hough circle transform in step 1 and performs the detection process again in the next position of the tableware by the moving of the conveyor; otherwise, it moves on to the next step. The detected tableware image is pattern-matched with the model image prepared in advance to improve accuracy further. If it does not pass, it returns to step 1 and performs the detection process again. If it passes, it is recognized that the detected position is correct. Then, the depth image determines the height of the tableware and the leftover food. Since the same tableware is not detected twice, a mask is attached to the position of the tableware and the mask is maintained until it comes out of the camera image at the speed v of the tableware. The distance from the end of view range to the robot is S, and the time from the tableware detected to arrive at the robot’s hand is ∆t. The robot’s hand closes and grabs the tableware when it arrives. Each part of the image processing are introduced in detail in Section 3.2 and Section 3.3.

### 3.2. Gray Image Processing

The position detection of the tableware was performed using a single channel gray image to reduce the amount of data processing.

**Figure 5:**
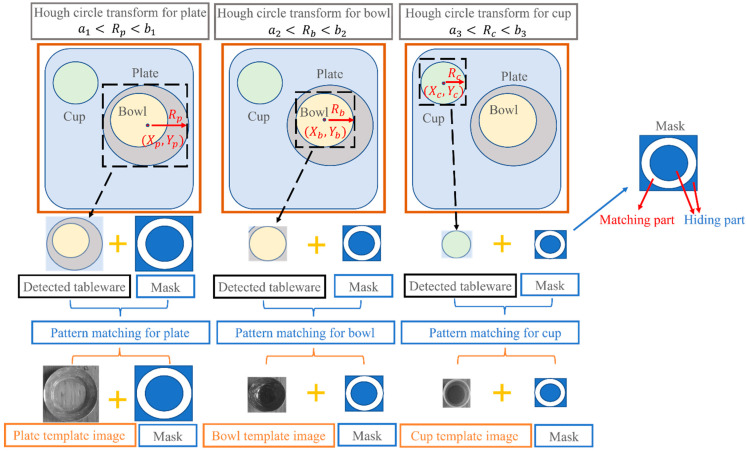
Image for each tableware detection.

Although this study aimed to detect overlapping tableware, the Hough circle transform cannot detect all tableware very well when the tableware overlaps [20], and the tableware that is not detected cannot be processed by the robot. To solve this, a dedicated detection for certain tableware was developed, as shown in Figure 5. Each image limits the radius, which guarantees that only specific tableware can be detected. Thus, even if the tableware overlaps, it can be detected. After tableware detection, the part of the tableware surrounded by the red dotted line, as shown in the figure, is removed, and pattern- matching with the model image was performed. If the degree of similarity is less than or equal to the threshold value, it is regarded as an erroneous test and excluded. It is recognized as the correct tableware position if it is beyond the threshold value. Then, a mask is attached to the detected position.

### 3.3. Depth Image Processing

**Figure 6:**
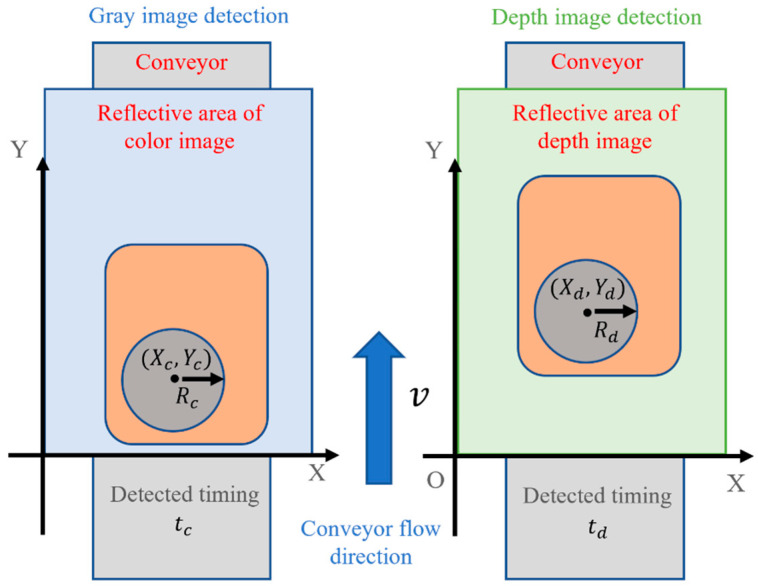
Coordinate determination.

The depth image was used to detect the tableware position through the Hough circle transform in the same way as the gray image. The gray image detects coordinates ($X_{c},Y_{c}$) and time $t_{c}$; depth image detects coordinates ($X_{d},Y_{d}$), time $t_{d}$, and the tableware moves at speed v. To prevent erroneous detection, ($X_{c},Y_{c}$), $t_{c}$, ($X_{d},Y_{d}$), and $t_{d}$ are checked to confirm if the coordinates are the same as shown in Figure 6.

Since the tableware moves only in the Y direction, it is recognized that the detected tableware position is correct if it is within the allowable position range ∆*b* as in Equation (2).

(2) $$\begin{array}{l} \left|X_{d}-X_{c}\right|<∆b\\ \left|(Y_{d}-\left(t_{d}-t_{c}\right)v)-Y_{c}\right|<∆b \end{array}$$

**Figure 7:**
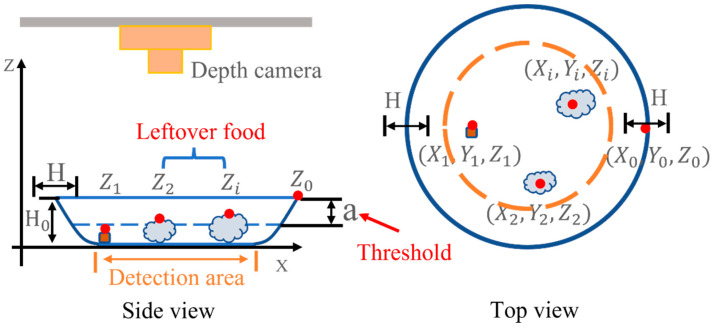
Edge height and leftover food detection.

After the tableware position was detected, the camera measured the height of the tableware’s edge, as shown in Figure 7. The initial edge of the tableware is the position away from the radius of the tableware from the center of the tableware obtained by the tableware detection. If only one point of the tableware edge detected circumference is measured, then there is a high possibility that the edge height measurement is wrong. Therefore, the height of the edge is measured within a specific width H determined by 1/2 of the radius of the tableware. Since the edges are higher than in a different place, the maximum value is considered and used as the height $Z_{0}$ of the edge of the tableware. The height $Z_{0}$ of the tableware’s edge is used as the standard for determining leftover food. When detecting leftover food, everything within the area drawn by the yellow dotted line, which the area of the tableware bottom measured in advance, is detected. A small amount of leftover food can be washed in the following process; therefore, leftover food is not considered. When the height is more than 5 mm, it is leftover, and a is the tableware height $H_{0}$ measured in advance minus 5 mm. The height of the conveyor is 0. If $Z_{0}-Z_{i}<a$, there is leftover food.

The movement of the conveyor belt changes the position of the tableware that makes the tableware to be detected many times by the camera, and the position of the tableware would be unknown. To prevent double detection, a dynamics mask is pasted with the same speed as the tableware to the detected tableware position after leftover food detection.

**Figure 8:**
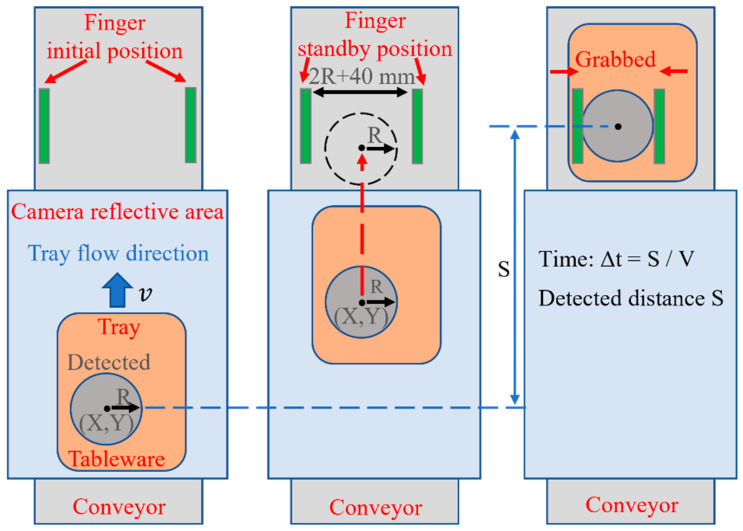
The method used to grab tableware.

After pasting the mask, the tableware is passed to the robot hand. The position information and diameter information of the tableware are provided by the camera. The robot hand keeps the distance between the fingers 40 mm larger than the diameter of the tableware and moves to the standby position to wait. The time ∆t for the tableware is calculated with distance S from the detected position to the grabbed position and the speed V of the conveyor belt. The robot closes the hand according to time ∆t and grabs the tableware, as shown in Figure 8.

## 4. Robot Prototype

**Figure 9:**
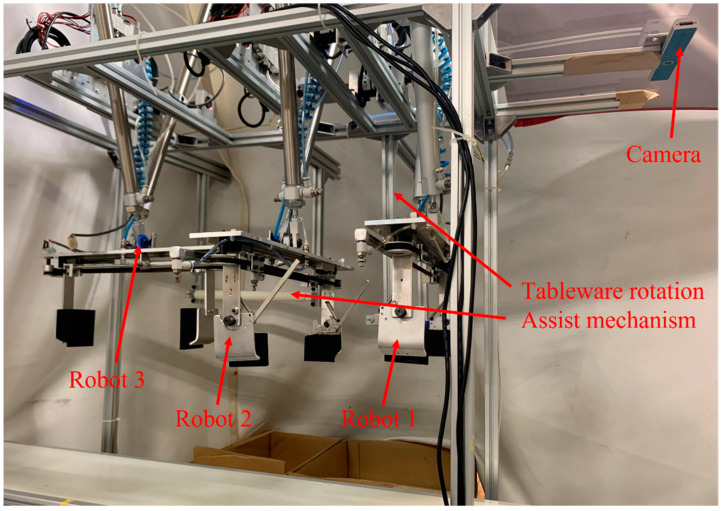
Robot prototype.

Figure 9 shows the tableware tidying up robot system. Robots 1 and 2 are type 1 robots, and robot 3 is a type 2 robot. The robot receives the information on the tableware detected by the camera, and the microcomputer calculates the required stroke of each air cylinder to the target position. The stroke is controlled by turning the solenoid valve ON/OFF at an air pressure of 0.3 MPa. The change in a stroke causes the angle of the air cylinder to change, and the angle of change is measured at any time with the encoder attached to the shaft. The solenoid valve is turned off when the target position is reached, and the robot stops. The size of the robot system is 1500 mm × 1000 mm × 1200 mm. The plate, bowl, and cup diameters are 160 mm, 103 mm, and 82 mm, respectively, and the heights are 20 mm, 55 mm, and 93 mm, respectively. The list of parts used is summarized in Table 2.

## 5. Image Processing and Tableware Sorting Experiment

### 5.1. Multiple Tableware Detection Experiment

**Figure 10:**
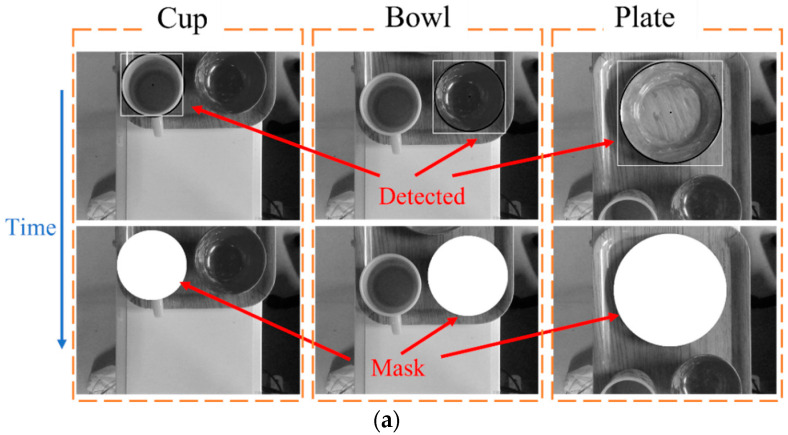

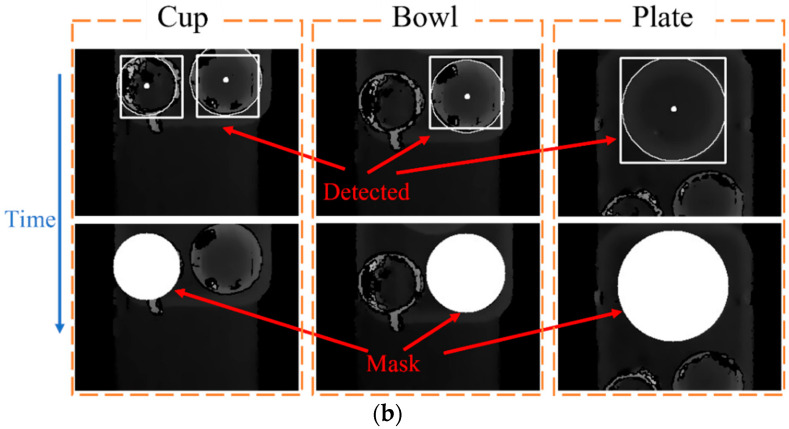
Multiple tableware image detection: (**a**) gray image detection; (**b**) depth image detection.

Experiments were conducted to verify the method of image detection. Three tableware samples were randomly placed on the tray flowing into the camera range on a conveyor at a speed of 15 mm/s. In order to detect tableware, the radius setting range of the Hough transform was plate: 77–88 mm, bowl: 48–67 mm, and cup: 42–50 mm in the gray image; plates: 75–93 mm, bowls: 50–68 mm, and cups: 37–62 mm in the depth image. The status of the image detection is shown in Figure 10, and the frame is the detected position of the tableware. Note that only the plate was detected in plate image detection. The detected position was not deviated, the same with the plate detection, and only the bowl was detected in the bowl image detection. However, in the cup depth image detection, a bowl was recognized as a cup due to erroneous detection of the depth image in this experiment. By determining whether the coordinates were the same as the position of the cup in the gray image, the erroneous inspection was eliminated. The whole process took 0.5 s.

### 5.2. Overlapping Tableware Detection Experiment

An experiment was conducted to verify the inspection method for overlapping tableware. There were mainly three situations: the bowl on the plate, the cup on the plate, and the cup on the bowl. If the bowl and plate overlap, the bowl was placed on the plate, and they were placed on the tray. The tray should flow into the camera range on the conveyor.

**Figure 11:**
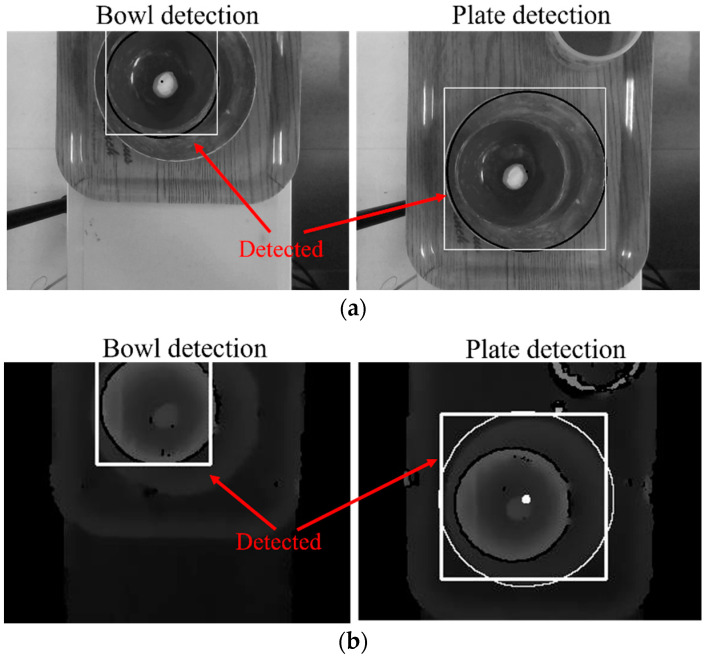
Overlapping tableware image detection: (**a**) gray image detection; (**b**) depth image detection.

Figure 11 shows the image detection results. The white frame indicates the position of the tableware. Since the tableware position of the gray image and depth image were the same, the tableware was detected correctly.

**Figure 12:**
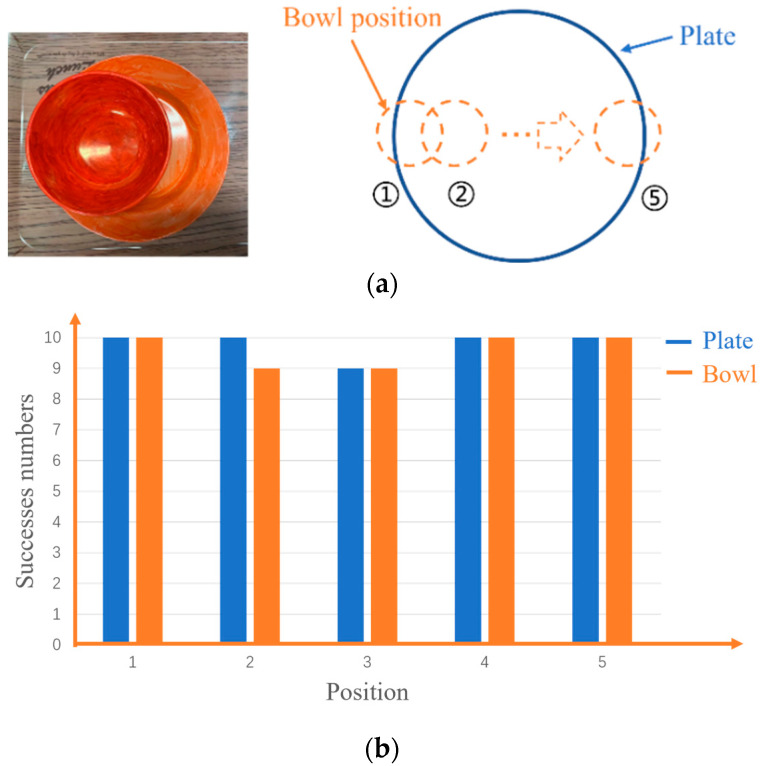
Detection of tableware overlap at different positions: (**a**) overlap of bowl and plate at different positions; (**b**) success rate of the overlap at different position.

In order to investigate the success rate when the bowl and the plate overlap at different places, as shown in Figure 12a, the detection rate is summarized in Figure 12b. Even if the overlapping position changes from the figure, the overlapping tableware can almost be detected. However, when the bowl is placed near the center of the plate, the bottom edge of the plate is too close to the edge of the bowl that affected the detection of bowls occasionally in the gray image. Since the depth image is unaffected by this factor, we considered only using the depth image to solve this problem.

**Figure 13:**
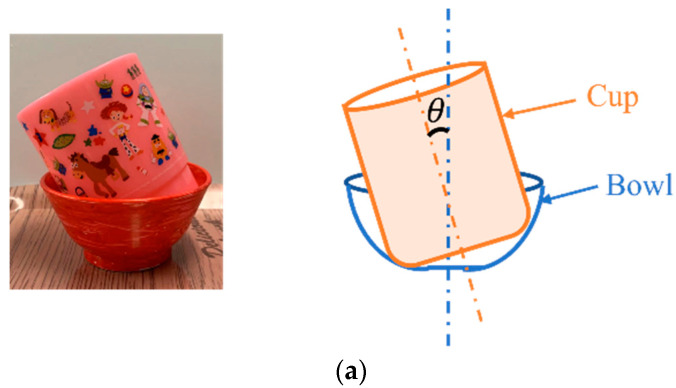

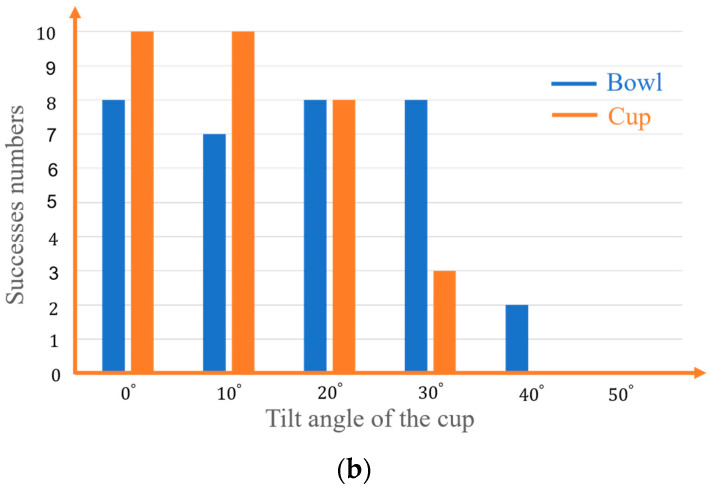
Detection of the tilt angle and success rate of the cup and the bowl overlap: (**a**) bowl and cup overlap; (**b**) success rate of tilt angles.

When the cup overlapped with the bowl, the bottom of the bowl was narrow and the cup could have tilted. In order to investigate the relationship between the tilt angle of the cup and the detection rate, the tilt angle was changed by 10 degrees from 0 to 50 degrees, as shown in Figure 13a, and the results are summarized in Figure 13b. For the cup, it could not be detected when the angle exceeded 30 degrees. The reason is that the edge of the cup looks elliptical as the angle increases in the image, making it undetectable. For the bowl, when it was larger than 30 degrees, it became almost undetectable. The reason was that the cup tilted at a certain angle and hid a part of the bowl that made the bowl not be detected. To solve it, the method that detected an ellipse was considered.

The image detection experiment of multiple and overlapping tableware was conducted 10 times, and the number of successes is summarized in Table 3. The tableware could be almost detected. The reason for the failure was considered to be that, when the tableware was placed at the corner of the tray, the corner affected the edge detection occasionally in the gray image. Since the depth image was unaffected by this factor, we considered only using the depth image to detect the tableware near the corner of the tray again.

### 5.3. Leftover Food Detection Experiment

An experiment was conducted to verify the leftover food detection method. The oil clay was used as leftover food that, with height $H_{i}=20\;\mathrm{mm}$, was put on a plate with height $H_{0}=20\;\mathrm{mm}$, and the plate was passed into the range of the camera on a conveyor. The threshold value of the leftover food assessment was 15 mm.

**Figure 14:**
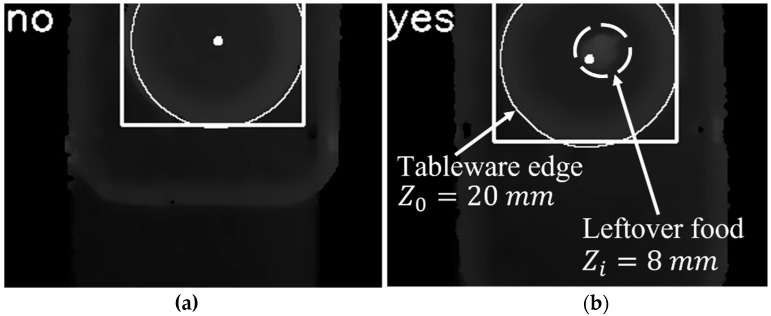
Leftover food detection by depth image: (**a**) there is no leftover food; (**b**) there is leftover food.

As shown in Figure 14, when there was no leftover food on the plate, a mark of no appeared in the upper left part of the image. When there was leftover food on the plate, it was detected that the tableware edge height was $Z_{0}=20\;\mathrm{mm}$ and the leftover food height was $Z_{i}=8\;\mathrm{mm}$. Because $Z_{0}-Z_{i}<a$, leftover food was judged, and a mark of yes appeared in the upper left part of the image. It was validated that this method of determining leftover food was effective.

**Figure 15:**
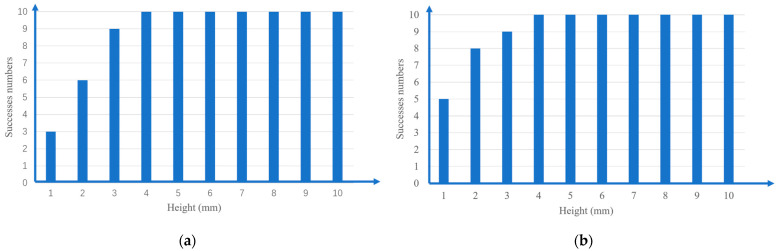
Detection of different heights of leftover food: (**a**) leftover food on the plate; (**b**) leftover food in the bowl.

In the leftover food detection experiment with the bowl and plate, the leftover food was placed on the plate and bowl at 1 mm intervals, using a height from 1 mm to 10 mm. Experiments were conducted 10 times each, and the results are summarized in Figure 15. The reason for the failure was an error of about 3 mm in the distance inspection, and when the height of the residual food size was less than 4 mm, there was an error in the judgment of the residual food due to the error of the distance inspection. A leftover food size less than 5 mm was not recognized as leftover food, and it could be washed with a dishwashing machine, so it was not processed. A leftover food size bigger than 5 mm being detected was the target in this study, and it could be appropriately detected.

### 5.4. Multiple Tableware Sorting Experiment

An experiment was conducted to determine if the robot could obtain the tableware information and tidy up the tableware. The conveyor moved at a speed of 15 mm/s, the air pressure was set at 0.3 MPa, and the tableware was randomly placed on the tray and passed to the robot by the conveyor. The higher tableware was tidied up. The cup was tidied up by robot 1, the bowl was tidied up by robot 2, and the plate was tidied up by robot 3.

**Figure 16:**
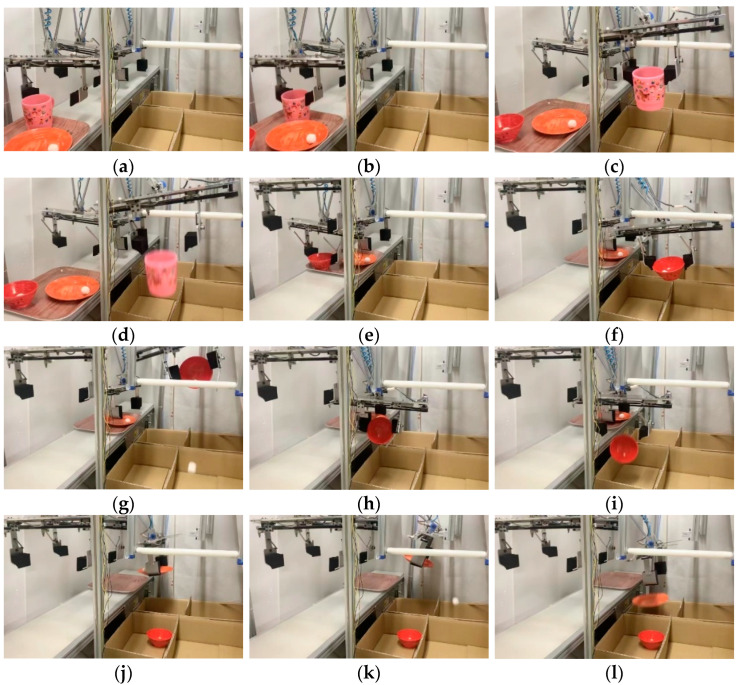
Multiple tableware sorting experiment: (**a**) hand standby; (**b**) cup grabbed; (**c**) moved to collection box; (**d**) cup thrown; (**e**) bowl grabbed; (**f**) moved to rotation assist mechanism; (**g**) leftover food thrown; (**h**) bowl thrown; (**i**) plate grabbed; (**j**) moved to rotation assist mechanism; (**k**) leftover food thrown; (**l**) plate thrown.

Each tableware item and leftover food were detected, as shown in (a) to (l) of Figure 16. Each corresponding robot hand went to the standby position, and the finger opened 40 mm larger than the tableware diameter. The fingers closed and grabbed the tableware at the moment when the cup reached robot 1’s hand. Since there was no leftover food in the cup, the fingers opened and dropped the cup into the cup collection box. At the moment when the bowl reached robot 2’s hand, the finger closed and grabbed the bowl. Since the leftover food in the bowl was also detected, the hand and the tableware were tilted by the rotation assist mechanism, and the leftover food was thrown away. After that, it was moved to the collection box of the bowl, and the bowl was dropped into the collection box. Similarly, when the plate reached robot 3’s hand, the fingers closed and grabbed the tableware. Since there was leftover food on the plate, the rotation part of the finger and plate was tilted with the rotation assist mechanism, and the leftover food was thrown away; then, the plate was dropped into the plate collection box. It took 57 s from the moment the first tableware was grabbed to when the last tableware was tidied up.

### 5.5. Overlapping Tableware Sorting Experiment

**Figure 17:**
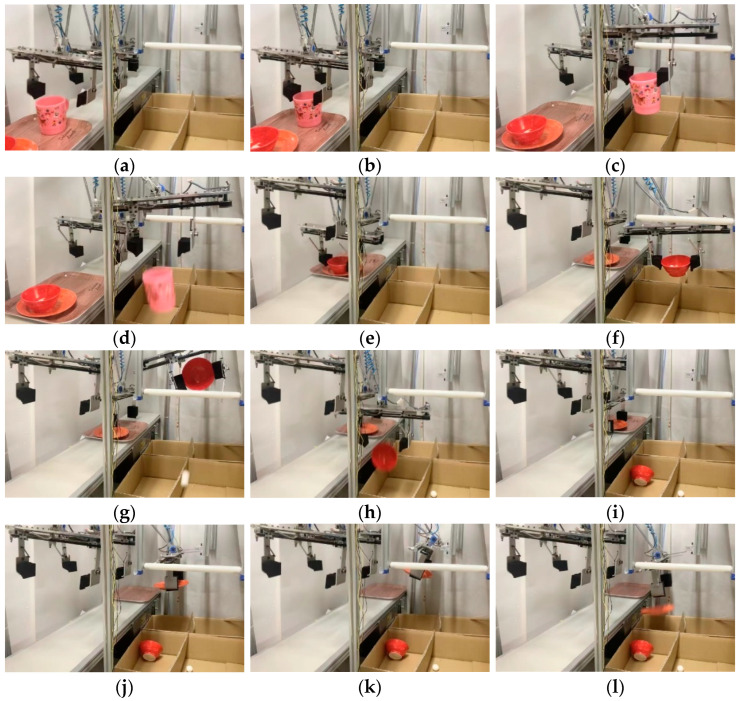
Overlapping tableware sorting experiment: (**a**) hand standby; (**b**) cup grabbed; (**c**) moved to collection box; (**d**) cup thrown; (**e**) bowl grabbed; (**f**) moved to rotation assist mechanism; (**g**) leftover food thrown; (**h**) bowl thrown; (**i**) plate grabbed; (**j**) moved to rotation assist mechanism; (**k**) leftover food thrown; (**l**) plate thrown.

The situations where tableware overlapped were mainly the plate and bowl, the plate and cup, and the cup and bowl. When the tableware was detected, as shown in Figure 17, the robot moved to the standby position. When the cup arrived at robot 1, the hand grabbed the cup and put it in the cup collection box. The overlapping plate had leftover food. Robot 3 grabbed the plate and threw away the leftover food, then dropped the plate into the collection box. Finally, the tray flowed into the tray collection box. It took 60 s to grab the cup and tidy up the plate into the collection box.

If there was no leftover food, the time from grabbing the tableware to dropping it into the collection box was 6 s, and if there was leftover food, it took 16 s for the type 1 robot and 15 s for the type 2 robot to tidy up, respectively. Each experiment was performed 10 times, and the number of successful experiments is summarized in Table 4.

The reason for the failure was that the distance between the tableware was too close, and there was no gap when the front tableware was grabbed and moved. Further, the back tableware was hit, the position was changed, and it failed when the robot grabbed it. To solve this, an auxiliary mechanism must be introduced to increase the tableware distance in the system. When the bowl and the cup overlap, the cup may be tilted due to the bottom of the bowl being small and failing when the hand grabs the handle of the cup. To solve this, a mechanism is introduced to rotate the cup.

## 6. Conclusions

We developed a tableware tidying up robot system that enables the processing of a large amount of tableware in a self-service restaurant at a high speed and low cost. The main contributions of this study are as follows:

(1) We were able to detect the position of the tableware at about 80% in both the normal state and the overlapping state of the tableware by combining the image processing of the gray image and the depth image using just one RGB-D camera, with which low cost was realized. It was also possible to determine whether there was leftover food in the tableware with the designed method using the depth function of the camera.
(2) By arranging multiple simple parallel robot arms side by side, the time of one tableware and three tableware with leftover food to be tidied up was about 16 s and 60 s at a pressure of 0.3 MPa, and the high-speed processing of tableware at a low cost was realized.
(3) In the tableware tidying-up experiment, it was confirmed that the designed robot mechanism can tidy up the tableware in the normal situation and the situation where the tableware overlaps, using the data from the image detection.
(4) It was confirmed that the two types of rotation mechanisms designed can throw away leftover food with the experiment of processing leftover food. The designed motorless rotation mechanism was able to achieve the goals of a lightweight robot hand and a low cost of the robot system.

Although the goals were achieved to a certain extent, achieving a processing speed of 12 s/tableware is still a challenge. To improve this, the air cylinder operating speed should be improved by increasing the air pressure and lightening the weight of the robot mechanism. Besides, the conveyor moves at a speed of 15 mm/s at this stage, and the processing speed of the robot system can be improved by increasing the speed of the conveyor belt and by increasing the number of robotic arms.

In the future, we will conduct detection experiments and tidying up experiments on chopsticks and spoons to enhance the research.

## Figures and Tables

**Table 1:** Final goals of the robot design.

Item	Value
System size	1500 mm × 1000 mm × 1200 mm
Processing speed	12 s/tableware
Robot moves range	800 mm × 200 mm
Positioning accuracy	20 mm
Tableware range	Diameter: 60–220 mm Height: 20–100 mm

**Table 2:** This table includes the main parts list of the robot system.

Parts	Manufacturer and Model Number	Specification
Air cylinder	SMC CM2YB20-600Z	Inner diameter: 20 mm stroke: 600 mm
Solenoid valve	SMC SY3320-5MZD-M5	$\mathrm{Flow}\;\mathrm{characteristics}:\;0.48{\;\mathrm{dm}}^{3}$/(s·bar) Number of ports: 5 Type of switching: 3
Encoder	Koyo Electronics Industries TRD-S1000A	Resolution: 1000 (PPR)
Camera	Inter SR305	Depth measurement range: 0.2–1.5 m Resolution: Color: 1920 × 1080 Depth: 640 × 480

**Table 3:** Accurate rate of tableware image detection.

Tableware Placement Status	Successes Times
Normal placement of plate, bowl, and cup	9/10
Cup and plate overlap	9/10
Bowl and plate overlap	9/10
Bowl and cup overlap	8/10

**Table 4:** Rate of sorting tableware without image processing failures.

Tableware Placement Status	Successes Times
Normal placement of plate, bowl, and cup	8/10
Cup and plate overlap	9/10
Bowl and plate overlap	8/10
Bowl and cup overlap	8/10

## Data Availability

Not applicable.
